# Spike Protein Fusion Peptide and Feline Coronavirus Virulence

**DOI:** 10.3201/eid1807.120143

**Published:** 2012-07

**Authors:** Hui-Wen Chang, Herman F. Egberink, Rebecca Halpin, David J. Spiro, Peter J.M. Rottier

**Affiliations:** Utrecht University, Utrecht, the Netherlands (H.-W. Chang, H.F. Egberink, P.J.M. Rottier);; and J. Craig Venter Institute, Rockville, Maryland, USA (R. Halpin, D.J. Spiro)

**Keywords:** virulence, pathogenesis, virus tropism, feline coronavirus, feline infectious peritonitis virus, FIPV, feline enteric coronavirus, FECV, sequence, mutation, diagnosis, viruses, coronaviruses, zoonoses

## Abstract

Coronaviruses are well known for their potential to change their host or tissue tropism, resulting in unpredictable new diseases and changes in pathogenicity; severe acute respiratory syndrome and feline coronaviruses, respectively, are the most recognized examples. Feline coronaviruses occur as 2 pathotypes: nonvirulent feline enteric coronaviruses (FECVs), which replicate in intestinal epithelium cells, and lethal feline infectious peritonitis viruses (FIPVs), which replicate in macrophages. Evidence indicates that FIPV originates from FECV by mutation, but consistent distinguishing differences have not been established. We sequenced the full genome of 11 viruses of each pathotype and then focused on the single most distinctive site by additionally sequencing hundreds of viruses in that region. As a result, we identified 2 alternative amino acid differences in the putative fusion peptide of the spike protein that together distinguish FIPV from FECV in >95% of cases. By these and perhaps other mutations, the virus apparently acquires its macrophage tropism and spreads systemically.

## Abstract 2

Mutations can occur erratically and accompany tropism changes, resulting in unpredictable new diseases.

Coronaviruses (subfamily *Coronavirinae*, order *Nidovirales*) are enveloped, plus-strand RNA viruses that infect mammals and birds. They are quite common and cause infections in humans and a wide variety of animals; infection typically results in respiratory or enteric disease. Severe acute respiratory syndrome coronavirus (SARS-CoV), which emerged suddenly in 2002 and caused severe acute respiratory disease in humans, is the most notorious coronavirus. SARS-CoV spread rapidly around the globe, infecting thousands and killing ≈800 persons. The virus presumably originated from bats and was transmitted to humans either directly or by using civets or raccoon dogs as intermediate hosts (*1**,**2*).

SARS-CoV best illustrates the remarkable potential for CoVs to change their tropism. Tropism switching has been implicated in the zoonotic emergence of human coronavirus OC43 from a bovine coronavirus and in turning transmissible gastroenteritis virus, an enteric porcine coronavirus, into porcine respiratory coronavirus, a respiratory pathogen (*3**,**4*). Such changes can be accompanied, although not necessarily, by cross-species transmissions; thus, the erratic occurrence and unpredictable new disease manifestations of tropism switching are a matter of public health concern.

The feline coronaviruses (FCoVs) present an example of pathogenetic change apparently associated with tropism switching. These viruses occur as 2 pathotypes with an enigmatic, even controversial, relationship: the low-virulence or nonvirulent feline enteric coronavirus (FECV) and the highly lethal feline infectious peritonitis virus (FIPV). FECV and FIPV are considered independently circulating viruses by some investigators (*5**,**6*). However, accumulating evidence supports the mutation hypothesis, which proposes that FIPV evolves from FECV by mutation in individually infected cats (*7**–**12*). A responsible mutation(s) has not been identified to back this hypothesis.

FECV is ubiquitous and spreads efficiently by the fecal-oral route; hence, seropositivity among cat populations can reach 90%, depending on the field conditions (*13*). The infection is restricted to the enteric tract, where the virus replicates in epithelial cells lining the gut mucosa. FECV infection is mild, causing transient enteritis that often passes unnoticed. The infection cannot be cleared efficiently by the immune system and thus persists, often for weeks or months, sometimes even longer (*14**–**18*). In contrast, FIPV is rare, but the consequences of infection are devastating. FIPV infection causes a progressive systemic disease called feline infectious peritonitis. The disease affects many organs, usually inducing fatal immunopathologic disease characterized by disseminated pyogranulomas and severe inflammatory damage to serosal membranes.

In the past, sequence differences in several virus genes, including those encoding membrane and spike (S) structural proteins and the so-called group-specific proteins 3c and 7b, have been implicated in the FCoV virulence shift (*5**–**7**,**10**,**11**,**19**–**22*). However, none of these differences appeared to consistently correlate with disease phenotype. To establish a consistent cause for a virulence shift in FCoV, specifically the predominant serotype I FCoV, we sequenced the entire genome of several FECV and FIPV specimens and then concentrated on the most conspicuous region of consistent difference by collecting and sequencing additional FECV and FIPV samples.

## Materials and Methods

### Viruses and Clinical Specimens

FECV strain RM and FECV strain UCD (FECV UU2) were propagated in specific pathogen–free cats. FIPV UU3 was obtained from a lymph node of a cat infected with FECV UCD; the presence of feline infectious peritonitis in the cat was pathologically confirmed. During 2006–2011, with the assistance of veterinarians in the Netherlands, we randomly obtained field cats with suspected feline infectious peritonitis and feces samples from apparently healthy cats from all geographic areas of the Netherlands; we did not use any selection criteria, such as age, sex, or breed of cat. Cats originated from 144 different catteries and single- and multicat households. Cats were pathologically diagnosed with feline infectious peritonitis by postmortem examination at the Veterinary Pathology Department, Utrecht University; findings confirmed that the cats had feline infectious peritonitis. Ascites samples and lesions from affected organs were obtained for RNA isolation. Fecal material from apparently healthy cats was obtained from the rectum by using a cotton swab.

### RNA Preparation

We suspended fecal specimens to a final concentration of 10% (wt/vol) in phosphate-buffered saline by vigorously vortexing the specimens. The supernatant was cleared (centrifugation for 10 min at 3,000 × *g*) and then used for RNA extraction. Following the manufacturer’s protocols, we used the QIAamp Viral RNA Mini Kit (QIAGEN, Valencia, CA, USA) to extract viral RNA from 140 μL of fecal supernatants or ascites and the QIAamp RNeasy Mini Kit to extract viral RNA from 30 mg of organ tissue homogenate.

### FCoV Detection and Serotyping

We tested RNA isolated from organs or ascites of cats with feline infectious peritonitis and from feces of apparently healthy cats for the presence of FCoV RNA by using a reverse transcription nested PCR (RT-nPCR) targeting the highly conserved 3′ untranslated region (*23*). Samples with results positive for FCoV were checked for the virus serotype by using an RT-nPCR targeting the S gene (*24*). Only samples positive for serotype I FCoV were included in this study.

We determined the sequence in the S gene region of interest by using an RT-nPCR. In brief, we synthesized complementary DNA by using an antisense primer (5′-CCCTCGAGTCCCGCAGAAACCATACCTA-3′) and superscript II reverse transcriptase (Promega, Madison, WI, USA) at 50°С for 1 h. We then performed the PCR by using *Taq* DNA polymerase (Promega) and specific primers (sense 5′-CAATATTACAATGGCATAATGG-3′, antisense 5′-CCCTCGAGTCCCGCAGAAACCATACCTA-3′) for the first reaction and specific primers (sense 5′-GGCATAATGGTTTTACCTGGTG-3′, antisense 5′-TAATTAAGCCTCGCCTGCACTT-3′) for the second reaction. PCR cycling conditions were 30 cycles at 94°С for 60 s, at 50°С for 30 s, and at 72°С for 1 min plus a 7-min extension at 72°С at the end of the reaction. All enzymes were used according to the manufacturer’s instructions. Primer pairs were expected to generate a 598-bp product for the first PCR run and a 142-bp product for the second run.

Representative PCR products were purified by electrophoresis in 2% agarose gel followed by extraction from the gel by using a gel extraction kit (QIAGEN) according to the manufacturer’s recommended instructions. Macrogen Inc. (http://dna.macrogen.com/eng/) sequenced the gel-purified DNA.

### Full Genome Sequencing

Two 96-well plates of degenerate primers (Technical Appendix) were designed from aligned reference genomes by using a computational PCR primer design pipeline. The pipeline was developed at the J. Craig Venter Institute (JCVI) to produce tiled amplicons with an optimal length of 550 bp, with 100-bp overlap to provide 6-fold sequence coverage of the genome. An M13 sequence tag was added to the 5′ end of each degenerate primer and was used for sequencing. Primers were arranged in a 96-well plate format, and all PCRs for each sample were performed in 2 plates. The primers used in this study are listed in the Technical Appendix.

Sequencing reactions were performed by using Big Dye Terminator (Applied Biosystems, Foster City, CA, USA) chemistry. Each amplicon was sequenced from both ends by using M13 primers, and sequencing reactions were analyzed by using a 3730 ABI sequencer (Applied Biosystems). Raw sequence data were trimmed to remove any primer-derived and low-quality sequence; gene sequences were assembled by using a viral assembly tool (www.jcvi.org/cms/research/software). Assemblies were edited computationally and manually. When insufficient underlying sequence information was obtained, the sample was entered into the secondary sequencing pipeline and reamplified by using existing primers or primers designed from the problematic sequence assembly itself. The reamplified sample was then sequenced again.

An RNA virus genome prediction program called VIGOR (Viral Genome ORF Reader, JCVI (www.jcvi.org/vigor) can decode many classes of viruses, taking into account virus-specific features, such as alternative splicing, internal open reading frames, and ribosomal slippage. This program was used to annotate de novo assemblies of coronaviruses sequenced at JCVI and also to validate newly assembled genomes during the finishing process. Last, we performed a quality control assessment and manually inspected the gene predictions before loading them into the annotation database at JCVI, from which they were exported in formats acceptable to the National Center for Biotechnology Information.

### Multiple-Sequence Alignment

The full-length and partial FCoV genomic nucleotide sequences we obtained were deposited in the National Center for Biotechnology Information database (www.ncbi.nlm.nih.gov). The sequence accession numbers of the full-length FCoV sequences used in this study are listed in Table 1. The GenBank accession numbers for the partial S gene sequences are JQ304323–JQ304518. Multiple-sequence alignments were constructed by using Clustal W (www.ebi.ac.uk/clustalw) with the Lasergene MegAlign (DNASTAR, www.dnastar.com/t-sub-products-lasergene-megalign.aspx) and MEGA4 (www.megasoftware.net) software programs. To identify key differences between FIPV and FECV, we analyzed their genomes and proteomes; for each nucleotide or amino acid position, we determined the rate at which FIPVs differed from all FECVs at that position. Phylogenetic analysis was performed by using features of the MEGA4 suite of programs. Phylogenetic trees of these sequences were obtained by using the neighbor-joining method. The bootstrap consensus tree, inferred from 1,000 replicates, was prepared; positions containing gaps and missing data were eliminated from the dataset.

**Table 1:** GenBank accession numbers for viruses for which the genomes were fully sequenced in a study to distinguish virulent from nonvirulent feline coronaviruses

Virus strain	Accession no.
Feline infectious peritonitis viruses	
UU3	FJ938061
UU4	FJ938054
UU5	FJ938056
UU8	FJ938055
UU9	FJ938062
UU15	FJ938057
UU16	FJ938058
UU17	HQ012367
UU21	HQ012369
UU24	HQ012370
UU30	HQ392472
Feline enteric coronaviruses	
RM	FJ938051
UU2 (UCD)	FJ938060
UU7	FJ938053
UU10	FJ938059
UU11	FJ938052
UU18	HQ012368
UU19	HQ392490
UU20	HQ392471
UU22	GU553361
UU23	GU553362
UU31	HQ012371

## Results

### Full Genome Sequencing

To identify the distinguishing difference(s) between the FCoV pathotypes, we initiated a full genome sequencing program of FECVs found in the feces of apparently healthy cats and of FIPVs found in organs or ascites of cats with pathologically confirmed feline infectious peritonitis. To obtain a more extensive analysis of the coronavirus genome, this sequencing program is still ongoing; however, after the sequences of 11 genomes of each pathotype were completed, we performed a comparative FECV–FIPV analysis, screening the genomes for nucleotide differences (Table 1). This was done by counting, for every nucleotide position, the number of FIPV genomes for which the identity at that position differed from that in all FECV genomes.

**Figure 1:**
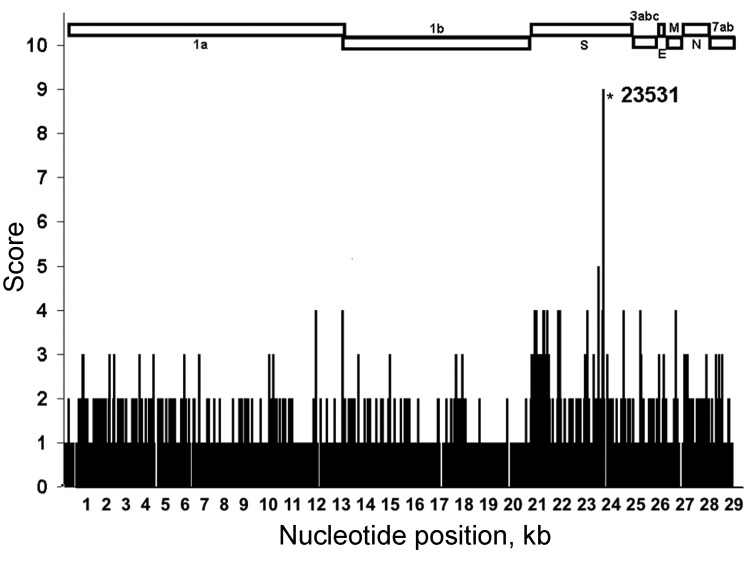
Comparison of full genomes of 11 lethal feline infectious peritonitis viruses (FIPVs) with full genomes of 11 nonvirulent feline enteric coronaviruses (FECVs). Nucleotide (nt) positions are shown on the x-axis; y-axis indicates number of FIPV genomes for which the identity at the nt position differed from identity at same position in all FECV genomes. FIPV strain C1Je (GenBank accession no. DQ848678) was used as the reference for nt numbering. *Highest difference score: 9 FIPVs had identities at nt position 23531 that differed from those at the same position in all FECVs. 1a, gene 1a; 1b, gene 1b; S, spike protein gene; 3abc, gene cluster 3abc; E, envelope protein gene; M, membrane protein gene; N, nucleocapsid protein gene; 7ab, gene cluster 7ab.

Our results showed that differences were scattered along the entire genome (Figure 1). At 2,963 (10%) of the 29,277 genome positions, the nucleotide identity in at least 1 of the 11 FIPVs did not occur in any of the 11 FECVs. Of these 2,963 positions, 1,187 occurred in gene 1ab, 1,246 in the S gene, 248 in gene cluster 3abc, 22 in the envelope protein gene, 42 in the membrane protein gene, 113 in the nucleocapsid protein gene, and 106 in gene cluster 7ab, showing the disproportionally large genetic variation in the S gene. The frequency with which differences occurred at different nucleotide positions across the genome showed the following distribution: a difference was detected 1× at 1,914 positions, 2× at 945 positions, 3× at 87 positions, 4× at 15 positions, and 5× at 1 position. At 1 position (23531), the nucleotide identity in 9 of the FIPVs was not found in any of the FECVs. No position(s) uniquely distinguished the 2 FCoV pathotypes.

Nucleotide identity differed the most at position 23531: it was highly conserved (100% A) in all FECV genomes, and it was C or T in 9 of the 11 FIPV genomes. This difference occurs in the S gene and results in an amino acid difference in the predicted S protein. Thus, although all FECV S proteins have a methionine at position 1058, a leucine is encoded in the 9 FIPVs, irrespective of the identity of the genetic difference (C or T).

### Sequencing of the S gene

**Figure 2:**
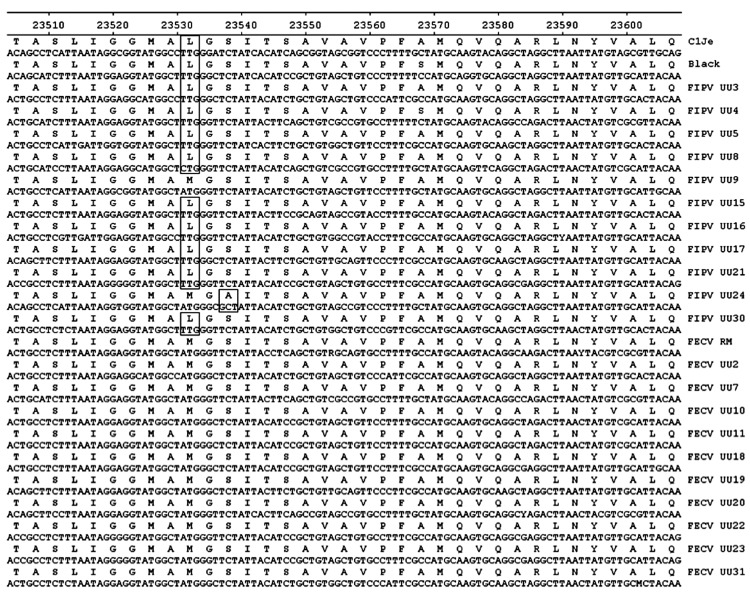
Alignment of partial nucleotide sequences and translated amino acid sequences in the spike protein of 11 strains each of 2 feline coronavirus pathotypes: FIPVs (lethal) and FECVs (nonvirulent). The viruses were sequenced in a study to distinguish virulent from nonvirulent feline coronaviruses (see Table 1). FIPV strain C1Je (GenBank accession no. DQ848678) was used as the reference for numbering. Sequence positions are shown along the top; virus strains are shown on the right. Specific differences between the pathotypes are boxed. FIPVs, feline infectious peritonitis viruses; FECVs, feline enteric coronaviruses.

To further investigate the single most prominent region of difference between FECVs and FIPVs, we established an RT-nPCR method to amplify and analyze the genomic region covering nucleotides 23442–24040 for the first PCR run and nucleotides 23451–23593 for the second run, which includes deviant position 23531. Altogether, 183 FECV and 118 FIPV RNAs isolated from different cats were sequenced in this specific region. Results for the 11 entirely sequenced FECVs and FIPVs are shown in Figure 2. The A at nucleotide 23531 was 100% conserved in all 183 FECVs in our collection. Of the 118 FIPVs, 96 (81.4%) had a T and 12 (10.2%) a C at this position; in both cases, this changes the methionine occurring at position 1058 in the FECV S protein into a leucine in FIPV (i.e., mutation M1058L).

### Phylogenetic Analysis

**Figure 3:**
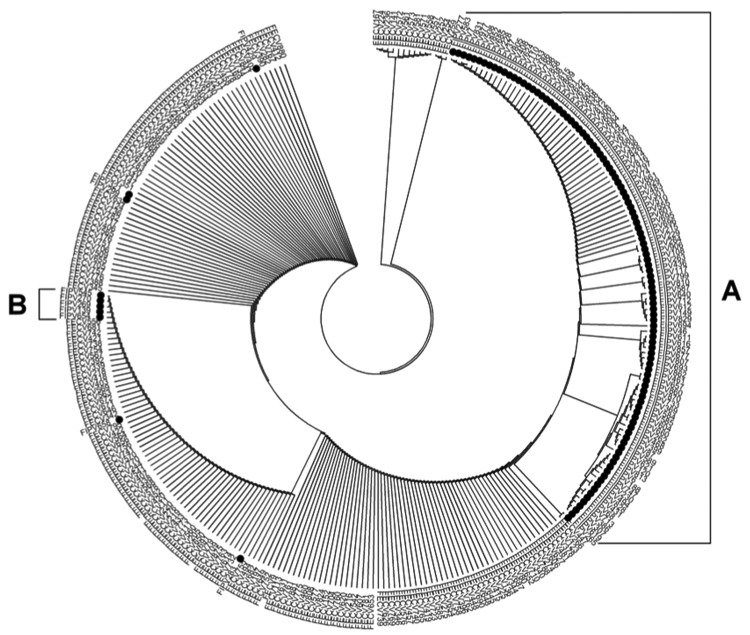
Phylogenetic tree based on partial amino acid sequences (aa 1056–1069) of the spike proteins of 118 feline infectious peritonitis viruses (FIPVs) and 183 feline enteric coronaviruses (FECVs) obtained by using reverse transcription nested PCR and sequencing of the distinguishing genomic region. A circular rooted neighbor-joining tree was constructed by using the bootstrap method and applying 1,000 replicates. Black dots indicate FIPVs. Clade A comprises FIPVs containing the M1058L mutation; clade B comprises FIPVs containing the S1060A mutation.

Assuming that the difference observed in 108 of the 118 sequenced FIPVs may be responsible for the virulent phenotype, the remaining 10 viruses should be expected to carry alternative differences. In search of those differences, we performed a phylogenetic analysis of the partial nucleotide sequences accumulated by the RT-nPCR procedure, but the results did not enable further differentiation (data not shown). When carrying out a phylogenetic analysis of the translated partial amino acid sequences, we observed a small but distinct second cluster B in addition to the major cluster A constituted by the FIPVs having leucine at position 1058 in their S protein (Figure 3). This smaller cluster, formed by 5 (4.2%) of the sequenced FIPVs, appeared to be characterized by the occurrence of an alanine at position 1060 (i.e., mutation S1060A), just 2 residues downstream of the M^1058^ that is changed in most FIPVs. All other FIPVs and all sequenced FECVs consistently had a serine at this position. The difference is brought about in all 5 cases by a T→G change at nucleotide 23537. Overall, we have detected characteristic differences with FECV for 113 (95.8%) of the 118 sequenced FIPVs. These differences were observed in both pathologic forms (i.e., wet and dry forms) of feline infectious peritonitis (Table 2).

**Table 2:** Prevalence of alternative mutations in feline infectious peritonitis virus spike protein of cats with wet and dry forms of FIP*

Mutation	Pathologic form of FIP	
	Wet	Dry
M1058L	71	46
S1060A	5	1
No mutation	3	3
Total	79	50

*Cats were diagnosed with the dry or wet form of FIP (feline infectious peritonitis) during postmortem examination by a veterinary pathologist. The occurrence and nature of the mutation in the spike gene was established by sequencing.

## Discussion

Our findings show differences in 2 alternative codons of the FCoV S gene that correlate with the feline infectious peritonitis disease phenotype in >95% of cases. Besides providing a realistic basis for diagnostic discrimination of the 2 FCoV pathotypes, our findings also support the mutation hypothesis. Thus, we propose that alternative mutations in the S protein of FECV give rise to a tropism change that allows the virus to escape from the intestine into body tissues, where it causes feline infectious peritonitis. Proof of this hypothesis will require introduction of these mutations into the FECV genome and demonstration of the virulence switch by infection of cats. However, this is a formidable challenge in the absence of a reverse genetics system and a proper cell culture system to generate and propagate these viruses.

Our findings relating the S protein to FCoV pathogenicity are not surprising, given earlier explorations into the involvement of various genes (7a, 7b, M, and 3c) (*5**–**7**,**10**,**11**,**19**–**22**,**24**,**25*). One of the most notable consequences of the presumed mutation in FECV is the acquisition of monocyte/macrophage tropism by the resulting virus (*26*). Thus, whereas replication of FECV is restricted to the epithelial cells lining the gut, the virulence mutation enables FIPV to efficiently infect and replicate in macrophages and spread the infection systemically (*26*). Such tropism change corresponds most logically with a modification in the S protein. An earlier study, using serotype II FCoVs, indicated a virulence role for the S protein (*21*); however, identification of the mutation(s) was not pursued because of the controversial nature of the FECV strain used in the study (*14*).

As for the serotype II viruses, the putative virulence mutations detected in the serotype I FCoV spike occur in the membrane-proximal domain of the protein. In coronaviruses, the S protein functions in cell entry; it is responsible for receptor attachment and membrane fusion. While the receptor binding site is located in the N terminal part of the protein, fusion is mediated by its membrane-proximal part. Coronavirus S proteins are class I fusion proteins, which typically contain domains instrumental for this process: 2 heptad repeat regions and a fusion peptide (*27*). The fusion peptide is located just upstream of the membrane-distal heptad repeat region, but it remains to be proven that it functions as a fusion peptide. The 2 putative virulence mutations identified in our study, M1058L and S1060A, map to this characteristic hydrophobic domain. Both changes are subtle and do not give clues as to their functional consequences. We assume, however, that these alternative mutations have a similar effect, and we speculate that the mutations in the remaining 4% of cases might also occur in the fusion peptide of the S protein.

If these mutations are all that is needed to convert a nonvirulent FECV into a lethal FIPV, the question arises as to why feline infectious peritonitis occurs so infrequently. For example, simple calculations based on a 10^−4^ frequency and a stochastic occurrence of RNA polymerase errors across the genome (*28*) predict that the M1058L mutation, for which 2 alternative substitutions of A^23531^ (to T or G) occur, would statistically arise once in every 1.5 × 10^4^ genomes produced. In experimental FECV infection of kittens, we showed that up to 10^8^ genome equivalents of the virus are shed per microliter of feces (*18*); thus, typical FECV infections would be expected to generate thousands of progeny carrying 1 of the critical mutations. However, the virulence phenotype supposedly associated with the mutation is not observed to any proportional extent. We can only speculate as to the reasons.

One likely possibility is that additional mutations (1 or more, perhaps alternative mutations) are required to generate the virulent pathotype. Such mutations would most probably involve the accessory gene 3c, which is intact in FECVs but severely affected in about two thirds of FIPVs (*7**,**10**–**12*). The 3c protein apparently is essential for replication of FECV in the gut but becomes nonessential once virulence mutation(s) elsewhere in the genome (e.g., in the S gene) enable the virus to infect macrophages and spread systemically. As we suggested earlier, loss of 3c function may not only be tolerated, it may even enhance the fitness of the mutant virus in its new biotope and, as a consequence, hamper its return to the gut. If the mutant virus is absent in the gut, it will not be shed in feces, providing an explanation for the seemingly rare incidence of feline infectious peritonitis outbreaks. Our discoveries of the critical differences between FECVs and FIPVs are clearly only a small step toward understanding the pathogenetic phenomena of feline coronavirus infections.

## Supplementary Material

Technical Appendix PCR primers used in a study to distinguish virulent from nonvirulent feline coronaviruses.
